# Fine Particulate Matter (PM_2.5_) upregulates expression of Inflammasome NLRP1 *via* ROS/NF-κB signaling in HaCaT Cells

**DOI:** 10.7150/ijms.46962

**Published:** 2020-09-01

**Authors:** Liu Dong, Ruiming Hu, Dandan Yang, Jinzhuo Zhao, Haidong Kan, Jianguo Tan, Ming Guan, Zhihua Kang, Feng Xu

**Affiliations:** 1 Department of Laboratory Medicine, Huashan Hospital, Shanghai Medical School, Fudan University, Shanghai, P. R. China.; 2 Department of Dermatology, Huashan Hospital, Shanghai Medical School, Fudan University, Shanghai, P. R. China.; 3 Shanghai Key Laboratory of Meteorology and Health, Shanghai Meteorological Service, Shanghai, 200030, China.; 4 Department of Environmental Health, School of Public Health and the Key Laboratory of Public Health Safety of the Ministry of Education, Fudan University, Shanghai, P. R. China.; 5 Key Laboratory of Public Health Safety of the Ministry of Education and Key Laboratory of Health Technology Assessment of the Ministry of Health, School of Public Health, Fudan University, Shanghai, P. R. China.; 6 Shanghai Key Laboratory of Meteorological and Health, Shanghai, P. R. China.

**Keywords:** PM2.5, Human Immortalized Epidermal Cells, Inflammasome, NF-κB, ROS

## Abstract

Skin, as the major organ of a human body, is constantly exposed to PM_2.5_ stimulation, which may exert specific toxic influences on the physiology of skin. This study aims to investigate the effect of PM_2.5_ on the formation of inflammasomes in skin cells and to explore the potential mechanism linking PM_2.5_ and skin inflammation. Changes in mRNA and protein levels of inflammasome-related genes were detected by real-time PCR and western blot in human immortalized epidermal cells (HaCaT) treated with PM_2.5_ at multiple concentrations for 24 hours. The expression of NLRP1 was increased significantly both in mRNA and protein levels after PM_2.5_ exposure while the elevated secretory protein level of IL-1β in cell culture was detected by ELISA, which is one of the main downstream factors of NLRP1. In addition, the upregulation of NLRP1 and IL-1β could be reversed by NF-κB inhibitor indicating that PM_2.5_ may promote NLRP1 expression through activating NF-κB pathway. Furthermore, high ROS level was also found in cells treated with PM_2.5_ and inhibition of ROS could also reverse NK-κB production stimulated by PM_2.5_ that means ROS is involved in this skin inflammation process.

## Introduction

Fine particulate matter (PM_2.5_) refers to particles with a diameter less than 2.5μm in aerodynamics which can gather toxic and harmful substances in the air. The compositions of haze pollutants are dominated by PM_10_, PM_2.5_ and NO_2_ in metropolis 1. Heavy air pollution may lead to acute symptoms in sensitive people, causing serious health damages and economic losses, and those affected residents are likely to show different degrees of clinical manifestations 2. The various components of air pollutants may probably have specific toxic effects on the skin. Particulate matters can directly enter the damaged skin, and organic compounds adhering to the particulate matters can also penetrate into the skin through a healthy skin barrier, resulting in systemic inflammatory reaction and skin damage 3. Studies have shown that acute PM_2.5_ exposure can reduce the activity of HaCaT cells and induce the release of inflammatory cytokines 4.

The inflammasome is an intracellular multiprotein complex involved in natural immunity and it consists of three components: a pattern recognition receptor (PRR), an apoptosis-associated speck-like protein containing a CARD (ASC), and an effector pro-caspase-1 5. PRR works as a cytosolic sensor (either a nucleotide-binding domain and leucine-rich-repeat-containing (NLR) protein or an AIM2-like receptor (ALR) protein) and ASC, a bipartite molecule, acts as an adaptor protein to bind NLRs or ALRs with the effector pro-caspase-1. After activation, caspase-1 cleaves pro-IL-1β and pro-IL-18, allowing them to mature into biologically active pro-inflammatory cytokines and induce pyroptosis subsequently 6. Most inflammasomes are composed of one or two NLR family members. Non-NLR proteins such as AIM2 and pyrin can also form inflammasomes 7.

Keratinocytes, main constituent part of epidermis, express antimicrobial substances in the event of injury or infection, and activate TLRs through PAMPs and DAMPs 8. All components required for the activation of inflammasomes are found in keratinocytes of psoriasis lesions, and NLR signaling genes NOD2 and PYCARD are upregulated in psoriasis affected epidermis. Activation of nuclear factor-kappaB (NF-κB) pathway signaling in psoriatic lesions promotes IL-1β and IL-18 production, and activates NLRP3 inflammasomes in keratinocytes 9. Studies have shown that caspase-1, caspase-5, and AIM2 inflammasome are also expressed in psoriatic keratinocytes 10-13. IL-1β level in serum is elevated in patients with vitiligo, and NLRP1 and IL-1β in vitiligo skin are significantly associated with the progression. By detecting NLRP1 markers, it can not only monitor the infiltration of inflammation during the progression of vitiligo, but also effectively assess the activity of the disease 14. In other skin diseases, such as contact dermatitis, skin cancer, acne, melanoma, eczema, and atopic dermatitis, there are evidences that inflammasomes participate in their pathophysiological processes 15.

## Materials and Methods

### Cell Culture

HaCaT cells were kindly provided by the Cells Center of Shanghai Institutes for Biological Sciences (Chinese Academy of Science, Shanghai, China). Cells were cultured in DMEM high glucose medium (Hyclone, USA) with 10% FBS (Gibco, Invitrogen) which regularly refreshed every two days in a 37 °C, 5% CO_2_ incubator and sub-cultured once every four days. The following experiments were performed in logarithmic growth phase cells.

### PM_2.5_ Collection and Cell Treatment

The PM_2.5_ particles were obtained, sterilized, and stored according to our previous study 4. After 24 hours of culture in 6-well cell plate at a concentration of 2 × 10^5^ cells/well, HaCaT cells were stimulated with various concentrations of PM_2.5_ when they were thoroughly spread. It is worth noting that PM_2.5_ particles must go through an ultrasonic vibration at least 90 minutes before use. PM_2.5_ was diluted in DMEM medium and the concentrations were set from 10 μg/mL, 25 μg/mL, 50 μg/mL, 75 μg/mL to 100 μg/mL with 0 μg/mL group as control. In the following experiments, inhibitors for NF-κB and ROS were used at a concentration of 20μM (JSH-23, NF-κB inhibitor) and 25μM (BAPTA, ROS inhibitor) 24 hours before PM_2.5_ exposure.

To obtain a better observation of cell morphology, crystal violet staining was used before microscope imaging (ZOE Fluorescent Cell Imager, Bio-Rad). Adherent HaCaT cells cultured in 6-well plate were washed twice with cold PBS and then incubated in room temperature with ice-cold 0.1% crystal violet solution diluted by methanol for fifteen minutes. After staining, cells were washed in deionized water several times and got dry at room temperature.

### Cell Viability Determination

HaCaT cells were pre-incubated in 96-well plates, 5000 cells per well the day before PM_2.5_ treatment. The next day, the medium was refreshed by new DMEM medium with PM_2.5_ at a concentration of 100 μg/mL and 0 μg/mL as control. Each group has three duplicate wells. Cell proliferation/cytotoxicity was measured by Cell Counting Kit-8 (Dojindo, Tokyo, Japan) after stimulation for 0 h, 24 h, 48 h, 72 h, 96 h and 120 h following the manufactory protocol. The cell viability was tested by measuring the optical density at 450 nm (OD450) with a Multiskan GO microplate reader (Thermo Fisher Scientific, USA).

### Quantitative Real-Time Polymerase Chain Reaction

Total cell RNA was extracted using RNeasy mini kit (Qiagen, Germany) according to the manufacturer's recommendations and quantified using a NanoDrop 2000 (Thermo Fisher Scientific, USA). PrimeScript™ RT reagent Kit (with gDNA Eraser) (TaKaRa, Japan) was used for the reverse transcription of cDNA. Relative quantitative RT-PCR (qRT-PCR) was performed with SYBR Premix Ex Taq II (TaKaRa) on an ABI PRISM 7500 Fast Sequence Detection System (Applied Biosystems, USA) according to the following parameters: 95 °C for 30 s, then 40 cycles of 95 °C for 5 s and 60 °C for 34 s and with 95 °C for 15 s, 60 °C for 1 min and 95 °C for 15 s. The primers used in qRT-PCR were shown in Table 1. The data were analyzed using the ΔΔCt method and β-actin was used as control.

### Immunoblotting Assays

The protein extracts from HaCaT cells treated by PM_2.5_ were obtained by cell lysis buffer (Beyotime, China). Total proteins for each sample were loaded onto a 10% sodium dodecyl sulfate polyacrylamide gel electrophoresis (SDS-PAGE) gel. After electrophoresis, proteins were transferred onto a PVDF membrane. After being blocked for 2 h at room temperature in 5% BSA (Beyotime, China), the PVDF membrane was incubated with primary antibodies over night at 4 °C.

The following primary antibodies were used in this research: anti-NLRP1 rabbit monoclonal antibody (1:1000, Cell Signaling Technology), anti-GAPDH mouse monoclonal antibody (1:1000, Proteintech), anti-IL-1-β rabbit monoclonal antibody (1:1000, Cell Signaling Technology), anti-NF-κB p65 polyclonal antibody (1:5000, Abcam). Then, the membrane was rinsed three times in TBST (10 min each at room temperature), and incubated for 1 h at room temperature with a HRP-linked secondary antibody of corresponding species (anti-rabbit IgG, 1:3000, Cell Signaling Technology; anti-mouse IgG, 1:3000, Cell Signaling Technology) and finally scanned with a LAS3000 imaging system (Fujifilm, Tokyo, Japan) after Immobilon Western (Millipore, USA) infiltration.

### Enzyme-Linked Immunosorbent Assay (ELISA)

To determine the concentrations of IL-1β, the culture supernatants of HaCaT cells under different treatment in 6-well plates were collected in every independent experiment. The level of extracellular IL-1β was determined using Human IL-1β/IL-1F2 Valukine ELISA Kit (Novus Biologicals, R&D Systems, catalog no. VAL101) according to manufacturer's instructions. The optical density of each well was determined by a microplate reader (Thermo Fisher Scientific, USA) at 450nm with 540nm as a wavelength correction. The concentration of IL-1β was calculated according to the standard curve. In three independent experiments, all samples and standards were assayed in duplicate and the mean values of the results were calculated.

### Cellular Reactive Oxygen Species (ROS) Detection

To detect the ROS level, HaCaT cells were cultured in 6-well plates under the treatment of 100 μg/mL PM_2.5_ for 24h. ROS was measured by DCFDA - Cellular Reactive Oxygen Species (ROS) Detection Assay Kit (Abcam, ab113851). 2',7' - dichlorofluorescin diacetate (DCFDA), cell permeant reagent, was used as a fluorogenic dye to indicate hydroxyl, peroxyl and other ROS activity within the cell. After staining, the ROS level of HaCaT cells was visualized by fluorescent microscopy (ZOE Fluorescent Cell Imager, Bio-Rad) and quantitated through flow cytometry (Accuri-C6, BD) measurement. At least 10,000 cells were analyzed per experimental condition in flow cytometry analysis, and mean fluorescent intensity (MFI) was used to calculate the fold changes between control groups and treated samples.

### Statistical Analysis

Statistical analysis was performed using GraphPad Prism7. The control group was compared with the patient group by independent t test. The difference was considered as statistical significance if *p*<0.05. All analyses were carried out three times independently.

## Results

### Cell proliferation was significantly inhibited after PM_2.5_ treatment with cell morphology largely changed

**Figure 1:**
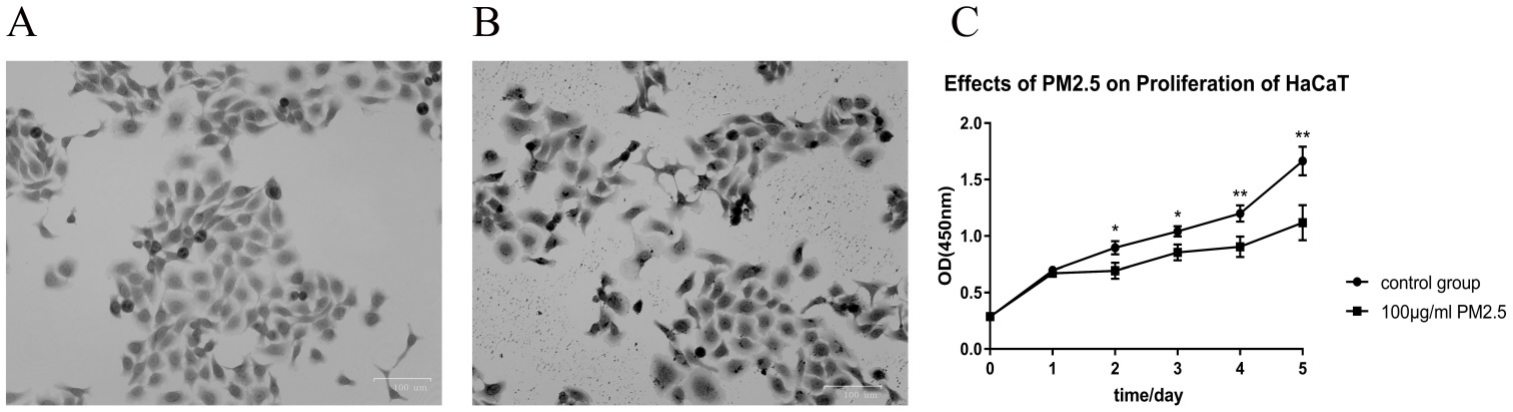
** Cell proliferation was significantly inhibited after PM_2.5_ treatment with cell morphology largely changed.** (**A**) 0.1% crystal violet staining of HaCaT cells. These cells were cultured in DMEM medium without PM_2.5_ treatment. (**B**) The cell morphology was changed after PM_2.5_ treatment (100 µg/mL) for 24 hours. (**C**) PM_2.5_ (100 µg/mL) could significantly inhibit the proliferation of HaCaT cells (*p<0.05; **p<0.01).

After treated with PM_2.5_ for 24 hours, a certain change in cell morphology was observed in HaCaT cells (Figure 1A and 1B). The cells were more irregular, the cell membrane structures were partly destroyed, and a large amount of PM_2.5_ particles penetrated the cells under the treatment of 100 μg/mL PM_2.5_. By 0.1% crystal violet staining, it was apparently that the cells swelled and the staining of the nucleus was deepened. PM_2.5_ may have a potential to penetrate the cell membrane and destroy its normal structure. We further quantified the effect of PM_2.5_ on the proliferation of HaCaT cells through detecting CCK-8 activity. The optical density of two groups (control group and PM_2.5_ treated group) was measured at 450 nm (OD450) at 0 h, 24 h, 48 h, 72 h, 96 h and 120 h. It was found that PM_2.5_ could significantly inhibit the proliferation of HaCaT cells (Figure 1C). Compared with the control group, there was a significant difference in the proliferation of cells in the treated group after 48 h (*p*<0.05), 72 h (*p*<0.05), 96 h (*p*<0.01) and 120 h (*p*<0.01).

### PM_2.5_ upregulated the expression of NLRP1 inflammasomes in HaCaT cells

**Figure 2:**
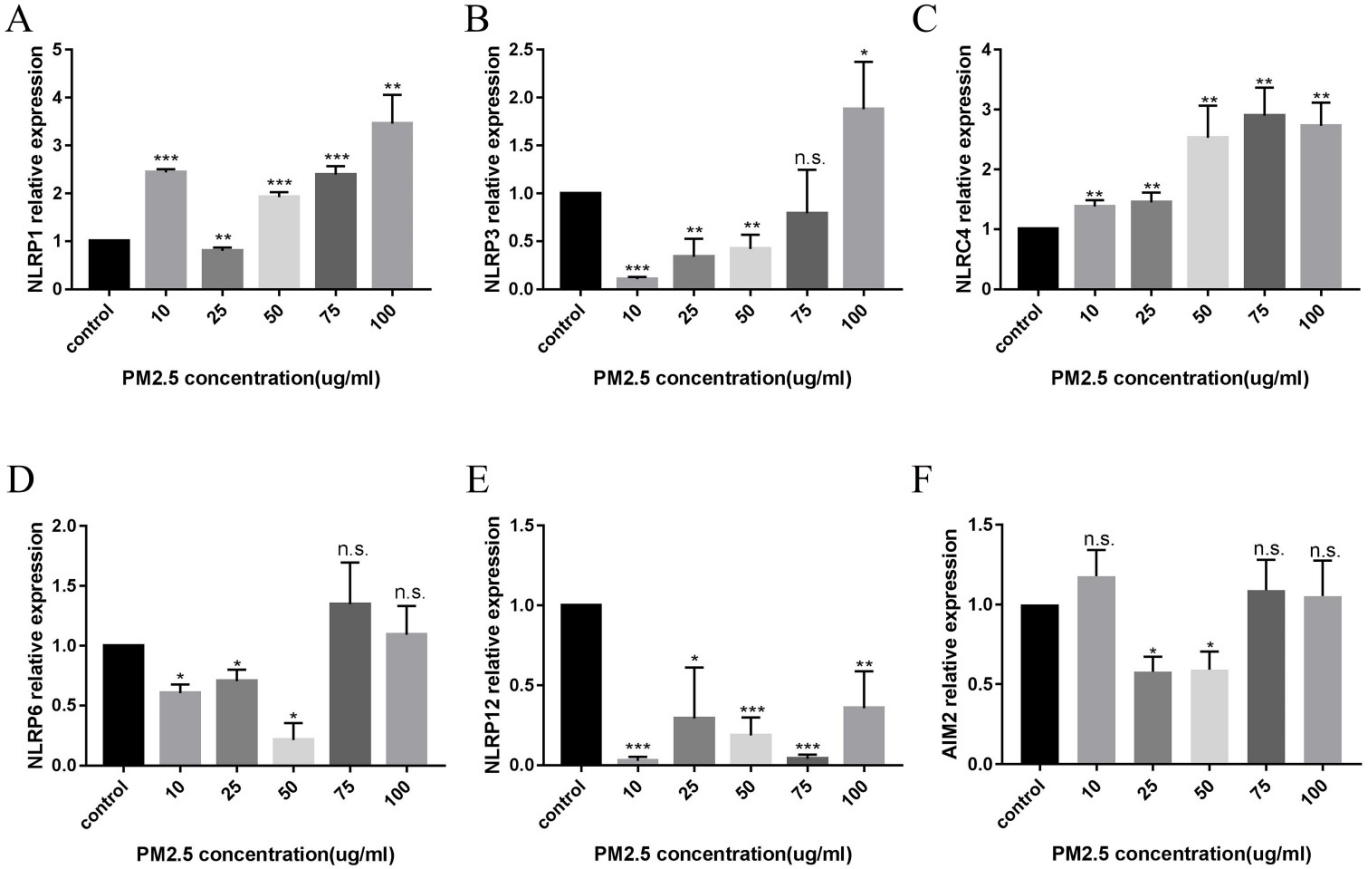
** The Expression of Inflammasomes mRNA after PM_2.5_ treatment was measured by qRT-PCR.** A total of six types of inflammasomes was measured in this study. (**A**) NLRP1 mRNA expression. (**B**) NLRP3 mRNA expression. (**C**) NLRC4 mRNA expression. (**D**) NLRP6 mRNA expression. (**E**) NLRP12 mRNA expression. (**F**) AIM2 mRNA expression (n=3, *p<0.05; **p<0.01; ***p<0.001; n.s. *p*>0.05).

**Figure 3:**
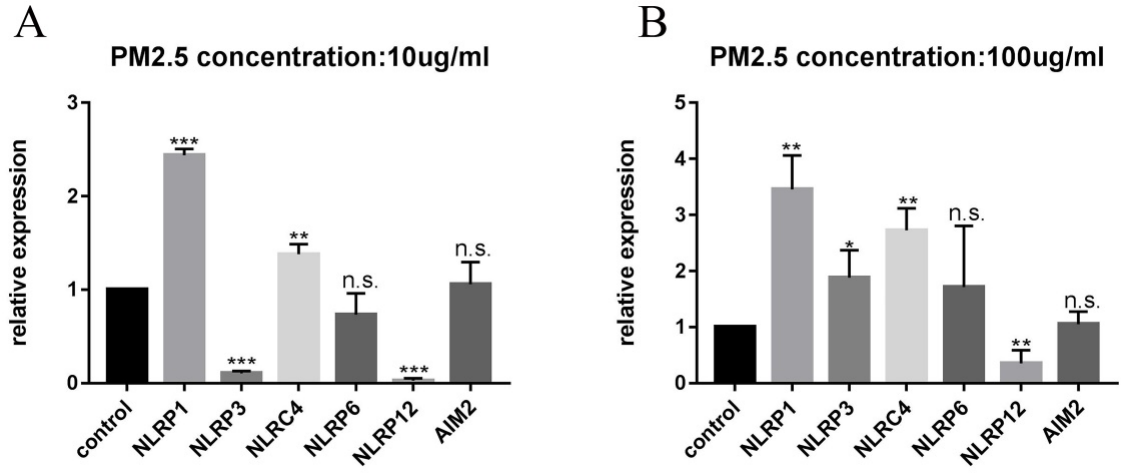
** Difference in mRNA expression of inflammasome at different PM_2.5_ concentration.** (**A**) The change of inflammasome at a concentration of 10 µg/mL. (**B**) The change of inflammasome at a concentration of 100 µg/mL.

After 24 hours of PM_2.5_ treatment, mRNA expression of inflammasomes in HaCaT cells was analyzed by relative quantitative qPCR. The changes of inflammasomes mRNA are shown in Figure 2. HaCaT cells stimulated by PM_2.5_ showed different mRNA expression levels in six inflammasomes related genes (NLRP1, NLRP3, NLRC4, NLRP6, NLRP12, and AIM2). The expression of NLRP1 and NLRC4 in HaCaT cells was significantly increased under the treatment of PM_2.5_, and the expression of NLRP12 inflammasome genes was decreased. The gene expressions of NLRP3, NLRP6 and AIM2 inflammasomes were not significantly changed. And it seems that there is an upward trend in all types of inflammasomes with the increase in PM_2.5_ concentration (Figure 3). Compared with the expression of PM_2.5_ at 10 μg/mL and 100 μg/mL, NLRP1 and NLRC4 were always elevated, and NLRP3 and NLRP12 increased slightly from the very low with the increase of PM_2.5_ concentration. The changes in NLRP6 and AIM2 were not obvious.

**Figure 4:**
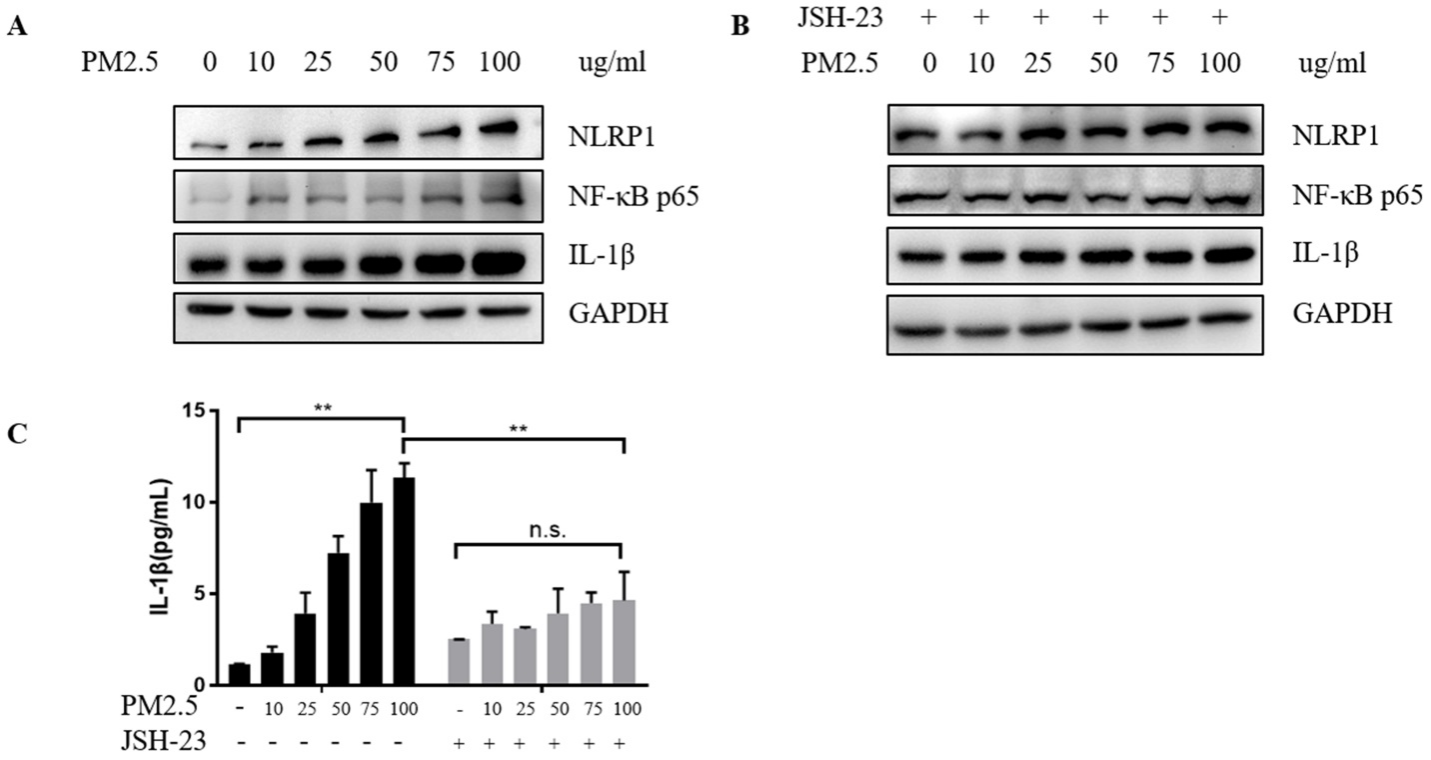
** PM_2.5_ upregulated the expression of NLRP1 inflammasomes in HaCaT cells.** (**A**) The protein level of NLRP1, IL-1β and NF-κB showed an increasing trend with the increase of PM_2.5_ concentration. (**B**) After JSH-23 (NF-kB inhibitor) treatment for 24 hours, there was no significant increase in the protein level of NLRP1, IL-1β and NF-κB. (**C**) The extracellular concentrations of IL-1β under different PM_2.5_ concentration with or without JSH-23 inhibition were measured by ELISA. The data were presented as mean±S.E.M. (**p<0.01; n.s. *p*>0.05).

After confirming the increase in mRNA level of NLRP1, we further verified the changes in protein level and found that the NLRP1 protein level showed a rising trend with the increase of PM_2.5_ concentrations (Figure 4A) as well as the level of IL-1β (Figure 4C) and at the same time, the level of NF-κB protein also elevated. While the cells were pretreated with 20 μM JSH-23 (NF-κB inhibitor) for 24 hours before PM_2.5_ exposure, NLRP1 and IL-1β level were no longer upregulated indicating that the activation of NLRP1 by PM_2.5_ may depend on NF-κB pathway (Figure 4B and Figure 4C).

### ROS was significantly activated in PM_2.5_ treated HaCaT cells

**Figure 5:**
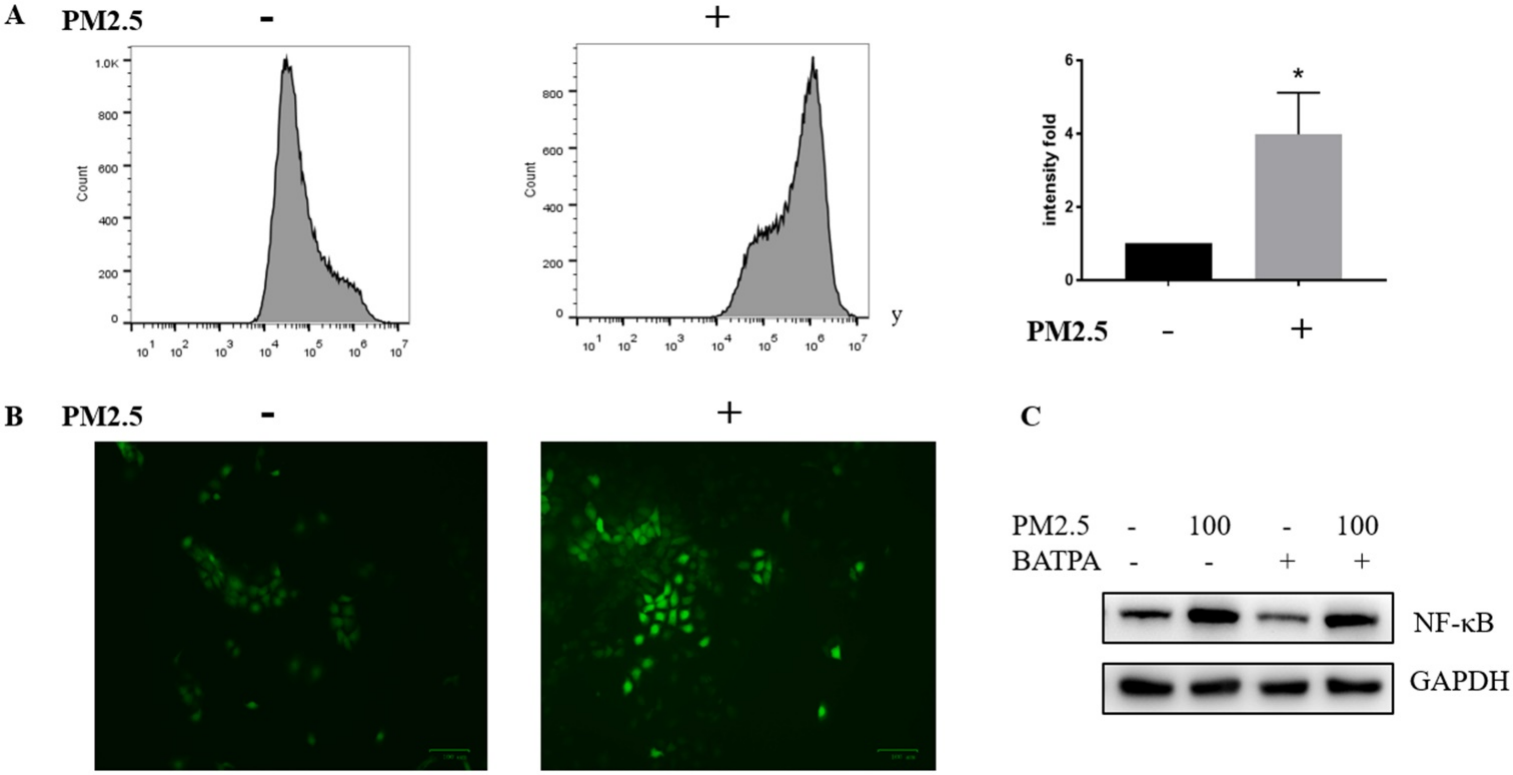
** ROS was significantly activated after PM_2.5_ treatment in HaCaT cells.** (**A**) HaCaT cells were exposed at 100μg/mL PM_2.5_ for 24 hours and ROS was detected by DCFDA - Cellular Reactive Oxygen Species (ROS) Detection Assay. Mean fluorescent intensity (MFI) was used as a parameter to demonstrate the fold change between control groups and treated samples. The increased intensity in ROS level was represented by a histogram. (**B**) According to manufacturer's protocol, ROS was also measured through fluorescence microscope after processing procedures mentioned above. (**C**) The control groups and treated groups were pretreated with or without 25uM BAPTA for 24 hours, and the protein level of NF-κB was significantly decreased after the inhibition of ROS.

We wonder how PM_2.5_ could activate NLRP1 via NF-κB pathway and tested whether ROS could act as a possible mediator of NF-κB activation. Reactive oxygen species (ROS) was significantly raised after PM_2.5_ treatment (100 μg/mL). Both fluorescent microscopy (Figure 5A) and flow cytometry measurement (Figure 5B) were used to determine the level of ROS which confirmed that ROS almost quadrupled after PM_2.5_ treated for 24 hours. Additionally, ROS inhibitor, BAPTA (25 uM) was used 24 hours before the PM_2.5_ treatment to pharmacologically inhibit ROS (Figure 5C). In this condition, reduced ROS resulted to a decrease NF-κB expression indicating a functional link with these two and the upregulation of NLRP1 might rely on ROS/NF-κB signaling in HaCaT cells.

## Discussion

PM_2.5_ promotes HaCaT cells to synthesize and release TSLP, TNF-α, IL-1α, and IL-8, and upregulates the expression IL-1β and IL-18 in the heart tissue in mice 4,16. Considering that several cytokines depend on the activation and maturation of inflammasomes, we supposed whether inflammasome is one of the products in skin cells upon PM_2.5_ stimulation. In our study, the gene and protein expression of NLRP1 is significantly increased in PM_2.5_ treated HaCaT cells.

Compared to the in-depth studies of NLRP3 inflammasomes, less is known about the biological functions of NLRP1. In skin tissues, the expression of NLRP1 is the highest while other inflammasomes including NLRP3 and AIM2 are not generally expressed 17. Both NLRP1 haploid genotypes and single nucleotide polymorphisms (SNPs) are associated with autoimmune disease, autoinflammatory disease and other diseases, including vitiligo and melanoma 18,19 . Mutations in NLRP1 proteins can activate inflammation pathway 17,20. We identified that PM_2.5_ could elevate the expression of NLRP1 both in mRNA and protein level. Previous studies have demonstrated that exposure to PM_2.5_ represents a high risk in the incidence of dermatitis and other skin diseases 21-23. Our study indicates that the increased NLRP1 might be the important protein involved in the PM_2.5_ induced skin inflammatory reaction instead of other inflammasomes.

In consideration of NF-κB pathway was closely related to the activation of NLRP1 and NLRP3 inflammasomes in neurons 24, we further investigated whether NF-κB would also play a role in skin inflammation. Our data showed that inhibiting the activation of NF-κB pathway could obviously suppress the rising expression of NLRP1 with the increasing concentrations of PM_2.5_. NF-κB is well-known as a multi-effect transcription factor which participates in immune regulation and inflammatory response. NF-κB-mediated gene expression can also regulate the production of inflammatory mediators 25. Franz et al 26 found that the signal provided by the NF-κB activator is essential for NLRP3 activation. So far, it has also reported that in atherosclerosis, triglycerides and VLDL-cholesterol contribute to the activation of NLRP1 through NF-κB motivation in endothelial cells 27. We demonstrated that the upregulated of NLRP1 by PM_2.5_ was dependent on the activation of NF-κB signaling which indicated that activated NF-κB pathway might play a key role in skin inflammatory reaction stimulated by PM2.5.

More than 50% of PM_2.5_ comes from industrial and vehicle exhaust emissions in Shanghai 28. Since the compositions of PM_2.5_ are extremely complicated, including various organic components, such as polychlorinated biphenyls (PCBs), 2,3,7,8-tetrachlorodibenzo-dioxin (TCDD) and polycyclic Aromatic Hydrocarbons (PAHs) 29,30. All these matters are able to produce reactive oxygen species (ROS) 31, which refers to those highly reactive, multi-electron, O_2_ containing molecules 32. Redundant ROS may also lead to a proinflammatory status and cause numerous serious diseases, particularly damaging hepatic, cardiovascular and pulmonary systems 33,34. Consequently, mitogen-activated protein kinase (MAPK) and NF-κB cascades are activated to balance the ROS formation and antioxidant activity 35. Here we show the significantly high ROS level in PM_2.5_ treated cells which may responsible for the activation of NF- κB signaling. A recent study also reported that in macrophages from zebrafish, ROS induced apoptosis and subsequently activated NLRP1 both *in vivo* and *in vitro*, while NLRP3 and AIM2 inflammasomes were barely upregulated in cell lines 36.

In conclusion, we clarify that PM_2.5_ activates NLRP1 via ROS/NF-κB signaling in HaCaT cells, which suggests that PM_2.5_ may induce dermatitis or increase the risk of other skin diseases through the inflammasome pathway.

## Figures and Tables

**Table 1:** Primers of inflammasomes (5'-3')

Gene	Forward	Reverse
NLRP1	GCAGTGCTAATGCCCTGGAT	GAGCTTGGTAGAGGAGTGAGG
NLRP3	GATCTTCGCTGCGATCAACAG	CGTGCATTATCTGAACCCCAC
NLRC4	TCAGAAGGAGACTTGGACGAT	GGAGGCCATTCAGGGTCAG
NLRP6	CCTACCAGTTCATCGACCAGA	CTCAGCAGTCCGAAGAGGAA
NLRP12	GGGGCTTGTCAGGAGATGG	AGTCCCTGGCATAGTAACCTC
AIM2	TGGCAAAACGTCTTCAGGAGG	AGCTTGACTTAGTGGCTTTGG
β-actin	AGTTGCGTTACACCCTTTCTTG	GCTGTCACCTTCACCGTTCC
